# *Eubacterium* sp. mediates the anti-obesity effect of lotus leaf extract via brown adipose tissue activation and white fat browning

**DOI:** 10.3389/fphar.2026.1727610

**Published:** 2026-03-09

**Authors:** Shuhan Zou, Xianmei Yuan, Zhihui Wang, Shang-Gao Liao

**Affiliations:** 1 State Key Laboratory of Functions and Applications of Medicinal Plants and School of Pharmaceutical Sciences, Guizhou Medical University, Guiyang, China; 2 University Engineering Research Center for the Prevention and Treatment of Chronic Diseases by Authentic Medicinal Materials in Guizhou Province, Guiyang, China; 3 Engineering Research Center for the Development and Application of Ethnic Medicine and TCM, Ministry of Education, Guiyang, China

**Keywords:** BAT, *Eubacterium* sp., lotus leaf, microbiota transplantation, obesity, WAT

## Abstract

*Nelumbinis* folium [Nelumbonaceae; *Nelumbo nucifera* Gaertn.] is a traditional Chinese medicine. Although studies have reported the efficacy of lotus leaf in weight management, the underlying molecular pathways necessitate further studies. This study aimed to elucidate the effects of lotus leaf on gut microbiota and their metabolites, especially long-chain fatty acids (LCFA), and measure how the effects of lotus leaf on gut microbiota help control obesity. The 12-week Lotus Leaf Extract (LLE) treatment mitigated weight gain, reduced liver and white adipose tissue mass, and activated brown adipose tissue in high-fat diet-induced obese mice, indicating enhanced energy metabolism. LLE reversed gut microbiota dysbiosis by enriching *Eubacterium* sp. in the gut. Microbiota transplantation indicated that *Eubacterium* sp. contributes to fat browning and thermogenesis, thereby alleviating host obesity, glucose homeostasis, dyslipidemia, and hepatic steatosis. In addition, *Eubacterium* sp. significantly increased the levels of margaroleic acid in mouse serum. In summary, the study found that lotus leaf extract can prevent obesity by modulating the gut microbiota, which in turn activates BAT activity and promotes the browning of WAT.

## Graphical Abstract

**Figure:**
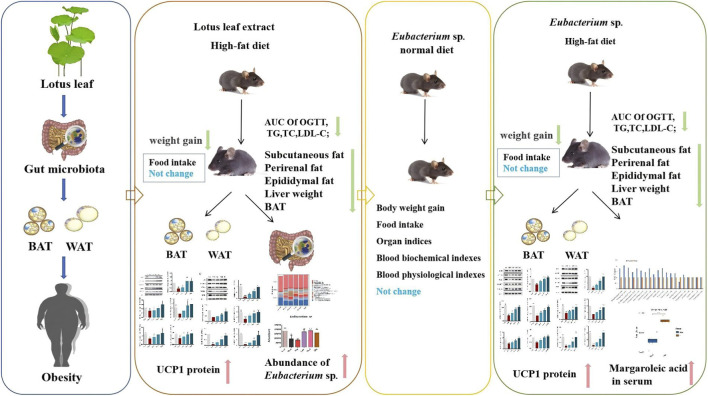

## 1 Introduction

Obesity is classified by the WHO as one of the top six contributors to the global burden of disease (Luo et al., 2020; Sung et al., 2019; Zhang et al., 2024; Irfan, 2024). As a major risk factor, obesity contributes to chronic conditions including cardiovascular disease, diabetes, and hypertension. Present anti-obesity interventions concentrate on decreasing energy intake and absorption. WAT and BAT are essential regulators of energy metabolism. Previous studies have shown that BAT facilitates weight control and health and offers an anti-obesity effect (Cheng et al., 2021a; Stanford et al., 2013). Heat production in BAT occurs predominantly through non-shivering thermogenesis, a process driven by lipid oxidation. This process is achieved through the upregulation of uncoupling protein 1 (UCP1), a protein located in the inner mitochondrial membrane, which is responsible for uncoupling respiration and thermogenic activity (Jung et al., 2019). Therefore, promoting BAT activity and inducing browning of WAT are considered promising molecular approaches for combating obesity (Cheng et al., 2021b).

The gut microbiota (GM) plays a crucial role in the host’s metabolic processes, functioning as an “energy-extracting organ” during the digestion and metabolism of nutrients, and serves as a key environmental factor influencing host energy balance (Cani and Hui, 2024; Wang et al., 2017). Changes in its composition and metabolites are closely associated with various metabolic diseases, including obesity, metabolic dysfunction-associated steatotic liver disease (MASLD), and type 2 diabetes, which are primarily characterized by alterations in the abundance of specific beneficial bacterial species (Qiu et al., 2021). Consequently, modulating the GM has emerged as a potential strategy for preventing and treating metabolic disorders. Additionally, studies have reported that the microbiota can ameliorate obesity-related metabolic disturbances by regulating brown adipose tissue (BAT) function (Iqbal et al., 2025; Roesler and Kazak, 2020). For example, research has shown that the probiotic *Lactobacillus* rhamnosus can improve obesity and related conditions, such as hyperlipidemia, by suppressing inflammation, activating BAT, and alleviating gut dysbiosis (Kang et al., 2022). Although clinically effective approaches for targeted GM modulation remain limited, advances in molecular tools such as metagenomics and metabolomics are advancing the understanding of complex host-microbe interactions, paving the way for novel strategies to prevent and treat diseases through precise regulation of microbial composition and function (De Vos et al., 2022; Hart et al., 2024; Kang et al., 2022).

For millennia, Traditional Chinese Medicine (TCM) has been practiced in clinical settings as a therapeutic approach and is valued for its low toxicity, reduced risk of dependence, and limited drug tolerance (Tang et al., 2021). Lotus leaf, native to China, has been utilized as a functional food and medicine in traditional healthcare and TCM for more than two millennia (Zheng et al., 2022). LLE has been reported to be applicable for obesity management. Recent studies have shown that it reduces hyperlipidemia and weight by inhibiting dietary fat/carbohydrate absorption and modulating energy use (Kim et al., 2019; Wan et al., 2022; Yan et al., 2017). LLE also upregulates PPAR-γ and leptin to promote lipid breakdown (Li et al., 2017). Furthermore, it activates the AMPK/DRP1/mitophagy pathway to induce white fat browning (Wang et al., 2024). In addition, a study has shown that LLE can regulate intestinal flora through dietary intervention and improve blood lipid metabolism (Zhang et al., 2023). However, the mechanisms through which lotus leaf prevents obesity by regulating gut microbiota remain unclear. Therefore, further studies are needed to explore the complex interactions linking lotus leaf, gut microbiota and their secondary metabolites, and obesity. To this end, we aimed to investigate the anti-obesity effects of LLE in HFD-fed mice, elucidate its impact on the gut microbiota and associated metabolites, and validate the causal role of the microbiota through microbiota transplantation experiments.

In the present study, we demonstrated that LLE significantly decreased adiposity by activating BAT. Additionally, LLE promoted the abundance of the intestinal *Eubacterium* sp., which generates margaroleic acid. Through microbiota transplantation experiments, we found that *Eubacterium* sp. counteracts obesity by enhancing BAT activity. Collectively, these findings suggest that *Eubacterium* sp. and its metabolite, margaroleic acid, might be used for preventing and treating obesity and related disorders.

## 2 Materiel and methods

### 2.1 Materials and reagents

Lotus leaf, *Nelumbinis* folium [species *Nelumbo nucifera* Gaertn. of genus *Nelumbo* Adans. of family Nelumbonaceae A.Rich] is one of traditional Chinese medicine recorded in the Chinese Pharmacopoeia (Chinese Pharmacopoeia Commission, 2025). The lotus leaf samples were purchased from Tongrentang Chinese Medicine store in Guiyang and authenticated by Dr. Rong-gui Qin (Guizhou Medical University). The voucher number for the plant (GZTM 0009013) was provided by Chinese Virtual Herbarium. The high fat feed (60% kcal fat, H10060) was supplied by Beijing Huafukang Biotechnology Co., Ltd. Orlistat capsules were sourced from Zhongshan Wanhua Pharmaceutical Co., Ltd. The antibodies including UCP1 (ab2413), ATP5A1 (ab19245), GAPDH (10025237), SDHB (00110734), UQCRC2 (00115767) and NDUFB8 (00110734) were purchased from Wuhan Sanying Biotechnology Co., Ltd. (China). Biochemical assay kits for triglycerides (TGs; 20241115), total cholesterol (TC; 20241113), high density lipoprotein cholesterol (HDL-C; 20241114) and low density lipoproteincholesterol (LDL-C, 20230316) were obtained from Nanjing Jiancheng Bioengineering Institute (Jiangsu, China). *Eubacterium* sp. (Strain ID: JCM 9976) was acquired from Ningbo Minghai Biotechnology Co., Ltd.

### 2.2 The preparation and chemical characterization of LLE

#### 2.2.1 Preparation of LLE

Dried lotus leaves were pulverized, mixed with 90% ethanol (1:8 w/v) for 1 h under the heat reflux method, and the guffs was extracted in 60% ethanol (1:8 w/v) for 1 h again. After completing the extraction, the filtrates were combined and concentrated, and finally freeze-dried to obtain the LLE, which was stored in a refrigerator at −20 °C. The extraction yield was 22.5%.

#### 2.2.2 The chemical characterization of LLE

The sample of LLE were determined employing UHPLC-Quadrupole - Time-of-Flight Tandem Mass Spectrometer (UHPLC-Q-TOF-MS/MS; Agilent 6520) with ACQUITY UPLC BEH C_18_ (2.1 × 100 mm, 1.7 µm) with 0.1% formic acid solution and acetonitrile as the mobile phase at a flow rate of 0.3 mL/min (30 °C). The chemical constituents of the LLE were profiled by UHPLC-Q-TOF-MS/MS in both ion modes. Accurate molecular weights were obtained to calculate elemental compositions, and structures were characterized by interpreting MS^2^ spectra. Compounds were identified by matching fragmentation patterns with literature data and established rules, with a mass error within 10 ppm. Isomers were distinguished using diagnostic fragments at different collision energies.The total ion chromatogram (TIC) of LLE is presented in Supplementary Figure S1. The detailed identification results for the individual compounds, along with their characteristic fragmentation patterns, are summarized in Supplementary Table S1.

### 2.3 Animals and experimental design

The Ethics Committee of Guizhou Medical University approved all animal experiments (SYXK (Gui) 2023-0002). All male C57BL/6J mice weighing 19–20 g were obtained from Beijing Huafu Kang Biotechnology Co., Ltd. (Production License: SCXK (Beijing) 2019-0010; Quality Certificate: 110324231105366441). Animals were housed at 23 °C–27 °C and 50%–60% humidity under a 12-h light/dark cycle. Food and water were available without restriction. Before starting the study on preventive treatment, each mouse was subjected to a 1-week adaptation phase. Body weight was measured weekly, and dietary consumption was documented every 2 days.

**FIGURE 1:**
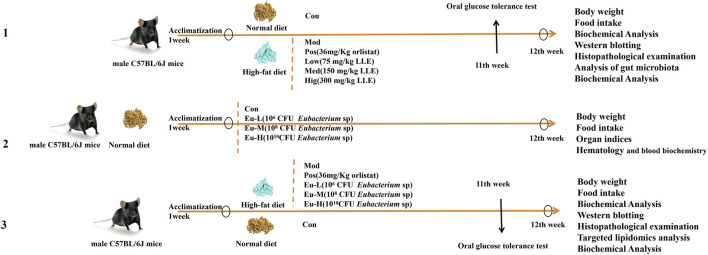
Schematic of the overall experimental design.

In this study, three separate experiments were conducted. This study examined the preventive role of lotus leaf extract (LLE) in high-fat diet (HFD)-induced obesity and identified differential bacterial species linked to its anti-obesity effects. A safety study was conducted to investigate the *in vivo* safety profile of these characteristic differential bacterial strains. resuscitated, and stored at −80 °C in 30% glycerol. Prior to use, the frozen stock was revived under anaerobic conditions (37 °C, 10% CO_2_) and passaged twice on solid GAM agar (Hope Bio-technology, HB8518-1). The activated bacteria were then grown in liquid GAM broth to late-log phase. Cells were harvested, washed, and resuspended in sterile PBS for oral gavage. All steps were performed in an anaerobic workstation to preserve viability. Furthermore, a validation study verified whether the characteristic differential bacterial strains directly activate BAT activity. Body weight and food consumption were recorded once per week. The schematic of the overall experimental design was shown in the Figure 1.

#### 2.3.1 Preventive study

The male mouse (*n* = 60) were randomly assigned to six groups. The control group (Con group, *n* = 10) was fed with conventional feed and the model group (Mod group, *n* = 10), the positive group (Pos group, *n* = 10) and the lotus leaf extract group were fed with high-fat feed. The Con group were given water daily. Mice in the pos group were given orlistat solution at a dose of 30 mg/kg. Mice in the low group were fed LLE solution at a dose of 75 mg/kg (Low group, *n* = 10) and mice in the medium group were fed LLE solution at a dose of 150 mg/kg (Med group, *n* = 10) and mice in the high group were fed LLE solution at a dose of 300 mg/kg (Hig group, *n* = 10). After 12 weeks of treatment, the mice were euthanized. Fecal material and blood were sampled. Blood was spun at 3000 rpm for 10 min, and the isolated serum fraction was frozen at −80 °C. Subsequently, their organs, including liver, subcutaneous fat (scWAT), perirenal fat, epididymal fat, white adipose tissue, and BAT, were collected and weighed.

#### 2.3.2 Safety study

A total of 40 C57BL/6J male mice (*n* = 40) were assigned randomly into four groups of 10 animals: Con group (Con, *n* = 10), 10^6^ CFU/day (Eu-L, *n* = 10), 10^8^ CFU/day (Eu-M, *n* = 10), and 10^10^ CFU/day (Eu-H, *n* = 10). After 12 weeks, mice were fasted for 12 h before blood collection and euthanized by exsanguination. The heart, liver, spleen, lungs, and kidneys were harvested, lightly blotted, and weighed. Organ indices were determined as organ mass (mg) per Gram of body weight. Blood samples were centrifuged (3,000 rpm, 10 min), and the serum fraction was frozen at −80 °C. All groups were maintained on a normal diet.

#### 2.3.3 Validation study

The mice were randomly distributed into six experimental groups: a normal diet (Con, *n* = 10), HFD (Mod, *n* = 10), HFD with 30 mg/kg orlistat (Pos, *n* = 10), HFD with 10^6^ CFU bacterial strains (Eu-L, *n* = 10), HFD with 10^8^ CFU bacterial strains (Eu-M, *n* = 10), and HFD with 10^10^ CFU bacterial strains (Eu-H, *n* = 10). Following the intervention, the animals underwent overnight fasting before euthanasia. Serum, liver, white adipose tissues, and BAT were meticulously collected and preserved at −80 °C until assay. Within a month, serum and tissue biochemical analyses were conducted to prevent unforeseen alterations in parameters.

### 2.4 Oral glucose tolerance test (OGTT)

One week before the end of the experiments, oral glucose tolerance tests (OGTT) were performed on mice from both the prevention study and the validation study. For the OGTT, mice were deprived of food for 12 h. A glucose solution (2 g/kg) was administered orally, and blood glucose was monitored from tail vein samples at five time points: baseline, 30, 60, 90, and 120 min. The corresponding AUC was determined for each animal.

### 2.5 Biochemical analysis

Serum concentrations of HDL-C, LDL-C, TG, and TC in mice from both the prevention and validation experiments were determined using commercial assay kits according to the manufacturers’ instructions.

### 2.6 Histopathological examination

Tissues (liver, perirenal fat, and BAT) were harvested from mice of the prevention and validation experiments and were then fixed in 4% paraformaldehyde for preservation. After fixation, they were embedded, cut into sections, and stained with hematoxylin and eosin (H&E). Tissue morphology was examined under a light microscope (MF53, Guangzhou Mingmei Optoelectronic Technology Co., Ltd.).

### 2.7 Western blotting

To investigate the impact of LLE and Eubacterium on both brown fat activity and white adipose tissue, Western blotting was conducted on mice in the respective experimental groups. Specifically, approximately 50 mg of scWAT and BAT were homogenized in 200 µL of RIPA lysis buffer and kept on ice for 30 min to ensure complete extraction. After centrifugation at 12,000 rpm for 30 min at 4 °C, the clear fraction was collected. Protein concentration was quantified using a BCA kit. Immunoblotting was then performed to detect UCP1, ATP5A1, SDHB, UQCRC2, and NDUFB8, with tubulin serving as the normalization control.

### 2.8 Analysis of gut microbiota

Fresh fecal samples were collected on the last day of the LLE treatment experiment to identify characteristic changes in the gut microbiota of mice. Fresh fecal DNA was isolated using the MagPure Soil DNA LQ Kit (Magen, China). The DNA was fragmented with a Covaris S220 system, then subjected to end-repair, adapter ligation, purification, and size selection. The V3–V4 region of the 16S rRNA gene was amplified with primers 343F (5′-TACGGRAGGCAGCAG-3′) and 798R (5′-AGG​GTA​TCT​AAT​CCT-3′). Purified PCR products were evaluated for quality with an Agilent 2100 Bioanalyzer (Agilent, United States), and sequencing was performed on an Illumina HiSeq system (Novogene, China).

Preprocessing of reads was conducted with fastp (v0.20.1) to remove adapters and low-quality bases. Reads were assembled using MEGAHIT (v1.2.9). Open reading frames of ≥500 bp were identified with Prodigal (v2.6.3). To generate a non-redundant catalog, sequences were clustered at 95% identity and 90% coverage with MMSeqs2 (v13.45111). Gene abundance was estimated with Salmon (v1.8.0). Functional annotation was carried out against NR, KEGG, COG, Swiss-Prot, and GO databases using Diamond (v0.9.10.111, e-value ≤1e-5). Taxonomic classification was based on NR to determine species abundance.

### 2.9 Hematology and blood biochemistry

At the end of the safety evaluation experiment. At the study endpoint, three mice from the control group and three from the high-dose group were randomly selected. Whole blood (approximately 1 mL) was drawn from the orbital venous plexus using aseptic technique and immediately placed into EDTA-K2 anticoagulated collection tubes. Complete blood counts (CBC) were analyzed with an automated hematology analyzer. Additionally, approximately 0.5 mL of blood was collected into anticoagulant-containing tubes, and serum biochemical parameters were measured using a fully automated biochemical analyzer.

### 2.10 Targeted lipidomics analysis

Serum samples were collected from mice on the final day of the validation experiment. Serum samples were combined with 480 μL of a methanol–acetone–water mixture (65:25:10, v/v/v) containing isotopically labeled internal standards (cholic acid-2,2,4,4-d4; palmitic acid-d3; [1-13C]-lauric acid). The sample was vortex-mixed for 30 s, then sonicated in an ice bath for 20 min. After centrifugation at 13,000 rpm for 10 min at 4 °C, 400 μL of the upper phase was removed and dried completely. The residue was dissolved in 240 μL of methanol:isopropanol (1:1, v/v) supplemented with 1 mM BHT, vortexed for 30 s, and sonicated again for 5 min in ice water. A second centrifugation step (13,000 rpm, 10 min, 4 °C) was performed, and the clarified extract was transferred to LC-MS vials and stored at −80 °C. Quantitative and qualitative assessment of metabolites was carried out using UPLC coupled to ESI-MS/MS.

### 2.11 Statistical analysis

Data are expressed as mean ± SEM. Analyses were performed using SPSS v26.0. Two-group comparisons were assessed by independent-sample t-tests, while multiple groups were evaluated with one-way ANOVA. Statistical significance was set at p < 0.05.

## 3 Results and discussion

### 3.1 LLE prevented HFD-Induced obesity in mice

**FIGURE 2:**
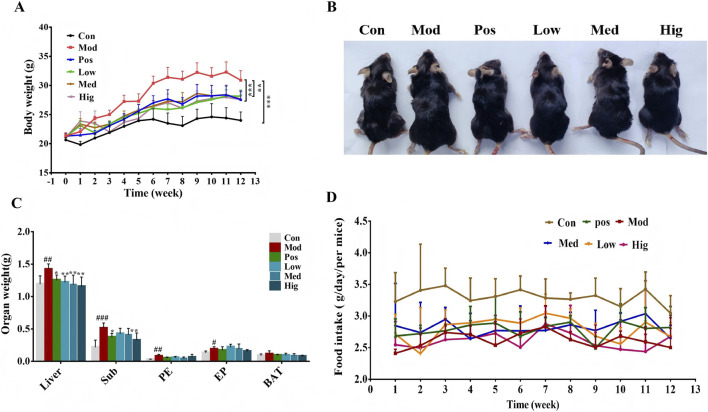
LLE supplementation ameliorated HFD-induced obesity in C57BL/6J mice (n = 6). **(A)** Regulatory effects of LLE on body weight, **(B)** Representative mice of Con, Mod, Pos, Low, Med, and Hig group; **(C)** Regulatory effects of LLE on weights of BAT, subcutaneous (Sub), perirenal (PE), and epididymal fat (EP) deposits, and liver, respectively. **(D)** Daily food consumption (in mice).

Mice fed an HFD were administered LLE at doses of 75, 150, or 300 mg/kg. Their obesity-related indices were compared with those of the Con and Mod groups. The Mod group displayed significant increases in body weight (Figures 2A,B; Table 1), liver weight, and adipose tissue mass (subcutaneous, perirenal, and epididymal fat) relative to the Con group (Figure 2C). After 12 weeks of treatment, LLE significantly attenuated HFD-induced elevations in body weight and liver weight. Treatment with LLE also markedly prevented adipose tissue accumulation, including subcutaneous, perirenal, and epididymal fat deposition, whereas the organ weight of BAT did not significantly change (Figure 2C). Notably, No significant difference in food intake among HFD-fed groups indicates that the effects of LLE were not mediated by reduced food consumption (Figure 2D).

**TABLE 1:** The effect of *lotus leaf* extract on body weight in HFD-fed mice (Mean ± SD).

Groups	Initial weight(g)	End weight(g)	Weight gain(g)	Weight gain rate (%)
Con	20.7 ± 0.3	24 ± 1.4	3.3 ± 1.7	16.32
Mod	21.3 ± 0.5	30.9 ± 1.6	9.6 ± 1.7***	44.92
Pos	21.3 ± 0.5	27.58 ± 1.47	6.2 ± 1.5***	29.43
Low	21.17 ± 0.2	28.17 ± 1.2	7.0 ± 1.1**	33.1
Med	21.07 ± 0.6	27.66 ± 1.1	6.6 ± 0.9***	31.37
Hig	21.08 ± 0.6	27.55 ± 1.9	6.4 ± 1.0***	30.75

***p < 0.001; **p < 0.01; *p < 0.05; *n* = 6.

### 3.2 LLE ameliorated lipid metabolism and blood glucose in HFD-fed mice

**FIGURE 3:**
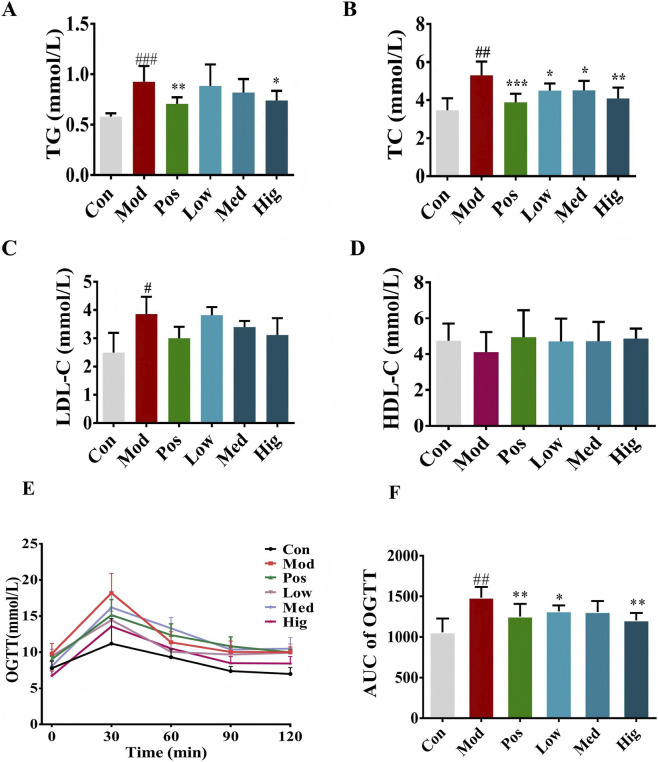
LLE supplementation reduced lipid profile and glucose tolerance in HFD-induced obese mice (n = 6).**(A)** total triglycerides (TG), **(B)** total cholesterol (TC) **(C)** low-density lipoprotein cholesterol (LDL-C), **(D)** high-density lipoprotein cholesterol (HDL-C), **(E)**, blood glucose, **(F)**, area under the curve (AUC) of oral glucose tolerance test (OGTT).

The role of LLE in modulating metabolism during obesity was evaluated by analyzing lipid and glycemic indices in the six groups. Compared with controls, HFD-fed mice displayed significant elevations in serum TC, TG, and LDL-C (Figure 3). Administration of LLE for 12 weeks markedly decreased these parameters relative to the Mod group (Figures 3A–C).

The OGTT results indicated that, 30 min after glucose administration, the Mod group displayed a pronounced increase in blood glucose, which was considerably greater than that of controls (p < 0.05). Glucose levels then gradually decreased over the next 90 min. AUC values were considerably higher in the Mod group relative to controls (p < 0.01). LLE treatment markedly improved glucose tolerance compared with the Mod group. The efficacy of LLE was comparable to that of orlistat (positive control) and exhibited a dose-dependent relationship (Figures 3E,F).

### 3.3 LLE prevented HFD-Induced liver steatosis and adipose hypertrophy

**FIGURE 4:**
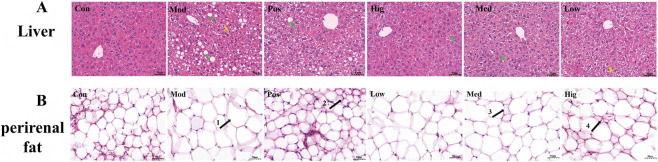
Effects of LLE on HFD-fed tissue damage and fat in mice. **(A)** H&E staining images of liver tissue, **(B)** H&E staining images of Perirenal fat.

Histopathological changes in liver and perirenal fat were assessed by H&E staining (Figure 4). In the Con group, hepatocytes were closely and neatly arranged, whereas the Mod group showed disorganized liver structure with extensive lipid vacuoles and severe degeneration. Adipose tissue sections revealed markedly enlarged and loosely arranged adipocytes in the Mod group compared with controls. LLE treatment improved hepatic architecture, reduced vacuolization, and restored hepatocyte organization with clearer cellular boundaries. It also improved the morphology of adipose tissue, reduced fat cell volume, making the liver and adipose tissue morphology of obese mice similar to that of the Con group (Figure 4A). Perirenal adipocytes were markedly larger in the HFD group than in the normal diet controls (Figure 4B, arrow 1). The positive drug orlistat and high-dose LLE significantly improved perirenal fat volume in mice subjected to HFD (Figure 4B, arrow 2, arrow 3).

### 3.4 LLE prevented HFD-Induced obesity by activating BAT and promoting white fat browning

**FIGURE 5:**
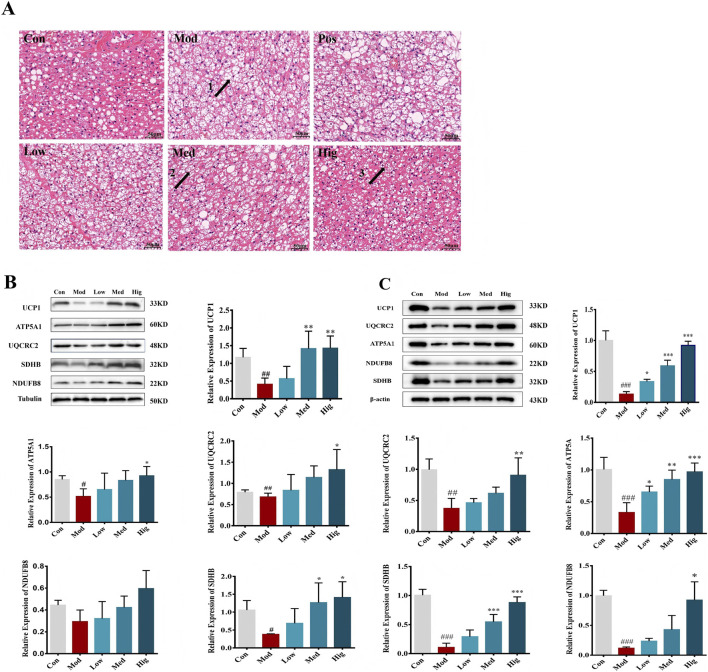
LLE supplementation promoted proteins’ expression associated with BAT. **(A)** H&E staining images of BAT, **(B)** proteins’ expression associated with BAT, **(C)** proteins’ expression associated with scWAT.

The molecular mechanism by which LLE controls weight gain in high-fat diet (HFD) mice was investigated. Tissue section analysis was performed. The results showed that adipose tissue cell volume was smaller in the Med and Hig groups (arrows 2 and 3). Additionally, adipose tissue whitening was significantly reduced (Figure 5A). To validate the effect of LLE on brown adipose tissue (BAT) activity, we analyzed key thermogenic genes at the molecular level. Specifically, we examined uncoupling protein 1 (UCP1) in BAT and subcutaneous white adipose tissue (scWAT), as well as the oxidative phosphorylation-related protein complex OXPHOS. As shown in Figures 5B,C, UCP1 and OXPHOS proteins were strongly upregulated following LLE treatment. These changes indicate that LLE can significantly enhance BAT activity, increase energy expenditure, and reduce body weight.

### 3.5 LLE upregulated the abundance of Eubacterium sp

**FIGURE 6:**
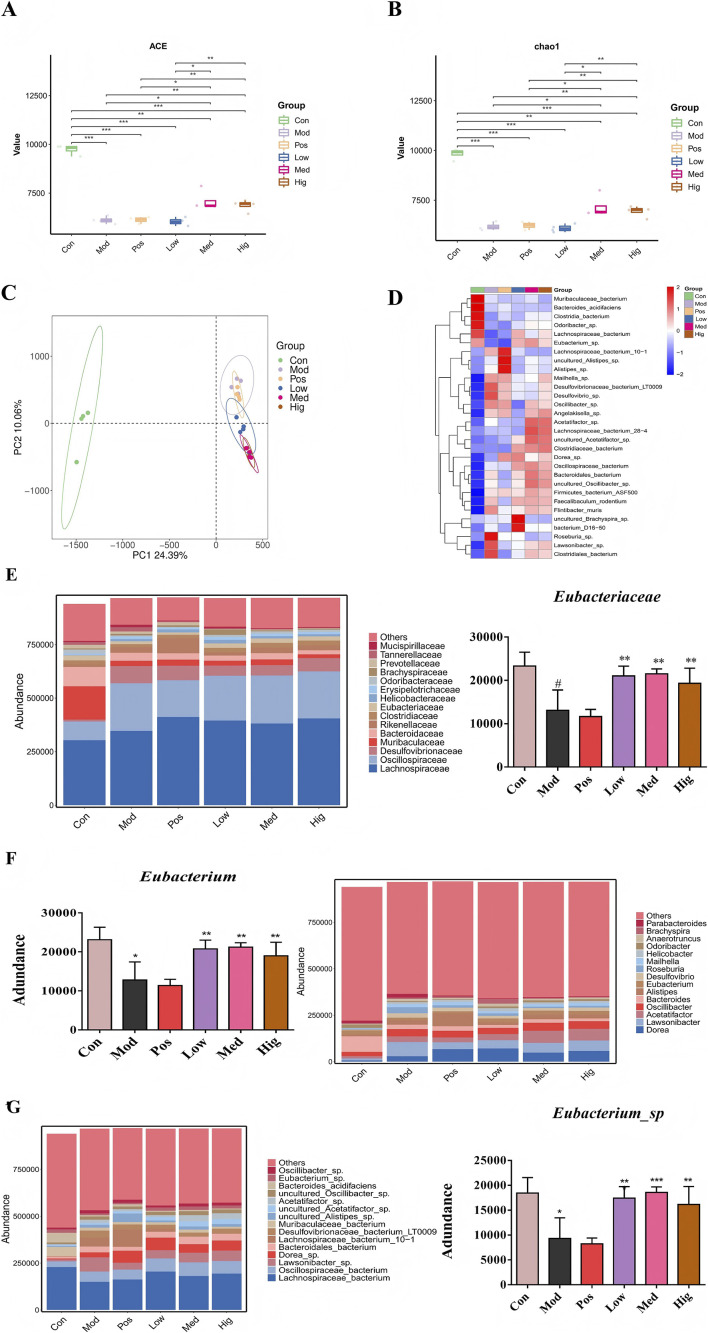
LLE alters microbiota composition in HFD-fed mice. **(A–B)** Alpha diversity index, **(C)** PCA analysis, **(D)** Hierarchically clustered heatmap, **(E–G)** Comparison proportion of family, genus and species levels of *Eubacterium* sp. in faeces detected.

Accumulating evidence established gut microbiota as a key regulator of obesity. Bacterial community diversity in fecal samples was evaluated through 16S rRNA amplicon sequencing to determine whether gut microbiota contributes to the preventive effects of LLE against obesity. LLE-treated mice showed significantly higher α-diversity values (ACE and Chao1) compared to the Mod group mice (Figures 6A,B). Using principal component analysis (PCA), β-diversity analysis exhibited distinct clustering patterns that clearly separated the six experimental groups (Figure 6C). At the species level, community heatmap analysis (Figure 6D) demonstrated a significant increase in the abundance of *Eubacterium* sp. in the LLE-treated group compared to the Mod group. The results of taxonomic analysis demonstrated a significant 2.2-fold enrichment (p = 0.000) of *Eubacterium* sp. at the family, genus, and species levels (Figures 6E–G). These results suggest that LLE significantly altered the composition of gut microbiota, with a marked increase in the abundance of *Eubacterium* sp (Supplementary Figure S2).

### 3.6 Eubacterium sp. did not affect normal growth in mice

**FIGURE 7:**
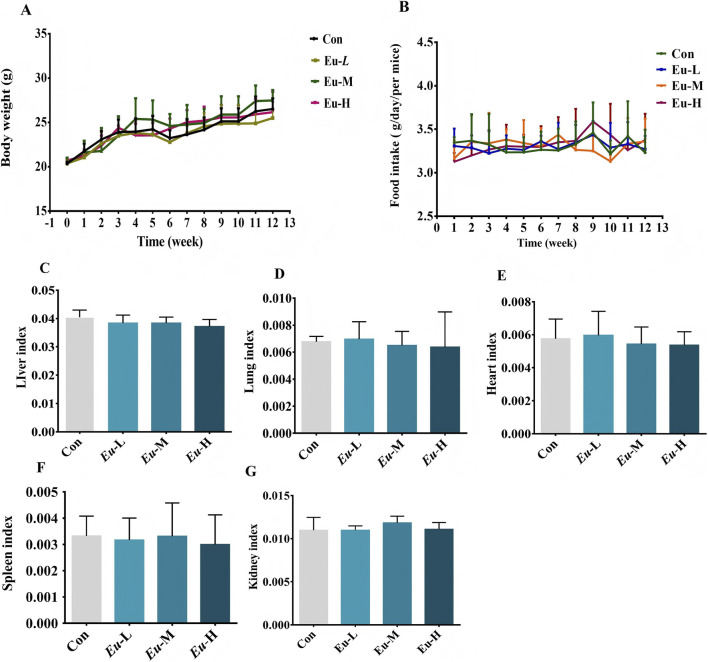
The effect of *Eubacterium* sp. on the food intake, body weight increment of mice and organ indices. **(A)** Average daily intake of mice. **(B)** Average daily weight gain. **(C–G)** organ indices.

Toxicological assessments often use body weight comparisons between experimental and Con groups as a sensitive measure for detecting organ changes caused by drug exposure. It was reported that certain *Eubacterium* species can metabolize in low-pH intestinal environments (Duncan et al., 2004). Moreover, the evidence confirms that oral gavage of live *Eubacterium* (10^6^–10^10^ CFU) results in a dose-dependent increase in its cecal abundance (Udayappan et al., 2016), verifying successful delivery of viable bacteria. In this study, after orally administering various concentrations of bacterial strains to mice, all groups exhibited good growth, behavior, and mental state, with no signs of toxicity or mortality. As illustrated in Figures 7A,B both food consumption and body weight increased in all experimental groups. However, the extent of weight gain did not differ significantly from that of the Con group (p > 0.05). Similarly, no group-to-group differences were detected in organ indices (Figures 7C–G)

### 3.7 Eubacterium sp. were not toxic to mice

Prior to evaluating its anti-obesity efficacy, we conducted an oral *in vivo* safety assessment of *Eubacterium* sp. in C57BL/6J mice, as its safety profile had not been previously established. Following 12 weeks of gavage administration at varying doses, all animals maintained good health with no mortality or overt signs of toxicity. Body weight gain and food intake remained comparable to the control group (p > 0.05). Moreover, organ weight indices—a well-established marker for toxicological evaluation (Nisha et al., 2009)—showed no significant alterations, indicating an absence of organ-level toxicity.

Hematological and serum biochemical analyses were performed to detect potential systemic effects, as changes in these parameters often precede clinical symptoms (Lu et al., 2021). At a dose of 10^10^ CFU, *Eubacterium* sp. did not induce significant deviations in biochemical indices (p > 0.05; Tables 2,3). Key hematological markers related to anemia (RBC, HGB, HCT, MCV, MCH, MCHC) and leukocyte subsets remained within normal ranges (Cascio and DeLoughery, 2017; Lu et al., 2021), further supporting the absence of hematological or metabolic disturbances.

**TABLE 2:** Changes of blood biochemical indexes.

Index	Unit	Con	Eu-H
Total protein (TP)	g/L	69 ± 4.83	67.23 ± 1.17
Albumin (ALB)	g/L	28.50 ± 2.90	34.57 ± 0.49^*^
Globulin (GLOB)	g/L	40.5 ± 3.37	32.67 ± 1.07
Total Bilirubin (TBIL)	µmol/L	3.48 ± 0.79	3.93 ± 0.61
Alanine Aminotransferase (ALT)	U/L	43.33 ± 11.05	45.00 ± 1
Aspartate Aminotransferase (AST)	U/L	195 ± 15.50	224 ± 10.21
AST/ALT ratio	​	5.31 ± 1.67	4.99 ± 0.33
γ-Glutamyl Transferase (γ-GGT)	U/L	1.2 ± 0.53	0.97 ± 0.23
Creatine kinase (CK)	U/L	2108.00 ± 162.30	2262.33 ± 33.47
Amylase (AMY)	U/L	839.33 ± 35.22	1075.00 ± 71.60^*^
Triglycerides (TG)	mmol/L	1.54 ± 0.18	1.12 ± 0.05
Cholesterol (CHOL)	mmol/L	3.47 ± 0.64	3.12 ± 0.08
Glucose (GLU)	mmol/L	4.5 ± 0.45	6.3 ± 0.84
Creatinine (CRE)	µmol/L	57.67 ± 23.86	63.66 ± 21.03
Blood urea Nitrogen (BUN)	mmol/L	8.60 ± 2.17	10.36 ± 1.34
BUN:Cr ratio	​	45.67 ± 17.46	43.67 ± 9.67
Total carbon Dioxide (tCO2)	mmol/L	17.67 ± 1.33	14.00 ± 0.58
Calcium (CA)	mmol/L	2.60 ± 0.19	2.26 ± 0.01
Phosphorus(P)	mmol/L	2.04 ± 0.13	2.36 ± 0.18
Ca × P product	mg/dL	65.33 ± 3.28	71.00 ± 6.56
Magnesium (Mg)	mmol/L	1.36 ± 0.08	1.31 ± 0.03
Alkaline Phosphatase (ALP)	U/L	55.00 ± 1.00	79.33 ± 4.62^**^

**p < 0.01; *p < 0.05; *n* = 3.

**TABLE 3:** Changes of blood physiological indexes.

Index	Unit	Con	Eu-H
Leukocyte count (WBC)	10^9^/L	4.42 ± 0.63	3.24 ± 0.27
Monocyte Percentage (Mon%)	%	6.09 ± 3.18	4.67 ± 0.21
Eosinophil Percentage (EOS%)	%	0.84 ± 0.59	1.40 ± 0.35
Basophil Percentage (BASO%)	%	0.07 ± 0.07	0.00 ± 0.00
Lymphocyte Count (Lymph#)	10^9^/L	1.86 ± 0.81	2.14 ± 0.26
Monocyte Count (Mon#)	10^9^/L	0.30 ± 0.19	0.15 ± 0.01
Eosinophil Count (EOS#)	10^9^/L	0.04 ± 0.03	0.05 ± 0.01
Basophil Count (BASO#)	10^9^/L	0.0037 ± 0.0037	0.00 ± 0.00
Nucleated red blood cell Percentage (NRBC%)	%	1.10 ± 0.49	1.78 ± 0.87
Nucleated RBC Count (NRBC#)	10^9^/L	0.05 ± 0.02	0.05 ± 0.02
Red blood cell Count (RBC)	10^12^/L	9.59 ± 0.60	9.37 ± 0.14
Hemoglobin (HGB)	g/L	162.33 ± 8.17	157.33 ± 3.28
Hematocrit (HCT)	%	47.43 ± 3.52	46.90 ± 1.30
Mean corpuscular Volume (MCV)	fL	49.37 ± 0.64	50.03 ± 0.72
Mean corpuscular Hemoglobin (MCH)	pg	16.93 ± 0.32	16.73 ± 0.20
Mean corpuscular hemoglobin Concentration (MCHC)	g/L	343.00 ± 10.40	335.±3.00
Red cell DistributionWidth-Coefficient of Variation (RDW-CV)	%	14.13 ± 0.23	13.90 ± 0.15
Red cell distribution width-standard Deviation (RDW-SD)	fL	22.10 ± 0.00	21.50 ± 0.30
Platelet Count (PLT)	10^9^/L	919.00 ± 38.00	891.00 ± 15.63
Mean platelet Volume (MPV)	fL	5.53 ± 0.09	5.80 ± 0.06
Platelet distribution Width (PDW)	fL	6.43 ± 0.20	6.93 ± 0.13

**p < 0.01; *p < 0.05; n = 3.

Collectively, these results demonstrate that orally administered *Eubacterium* sp. is well-tolerated in mice, supporting its safety for further investigation as a probiotic candidate. Future studies incorporating histopathological examination will provide a more comprehensive toxicological characterization.

### 3.8 *Eubacterium* sp. prevented obesity

**FIGURE 8:**
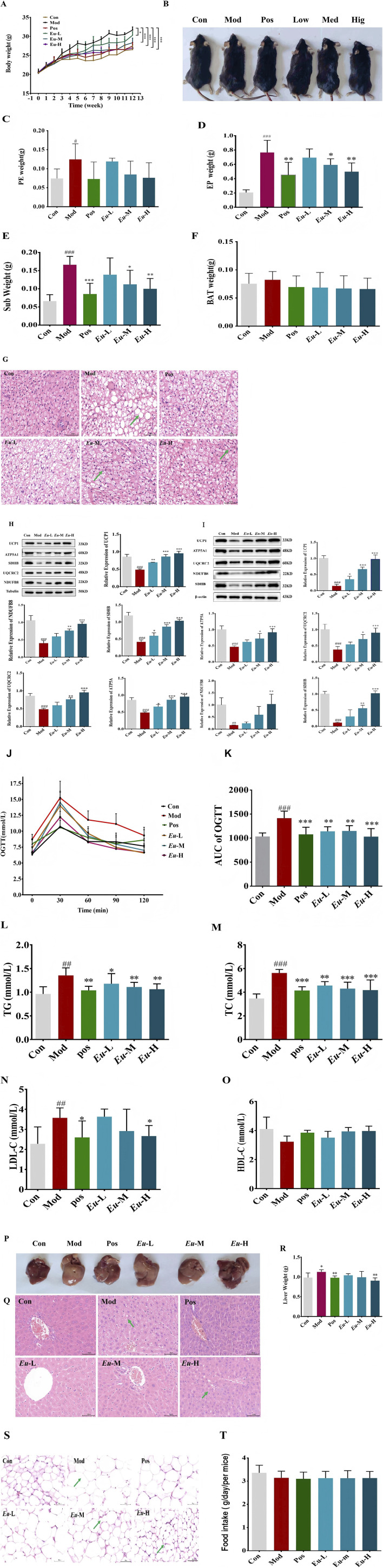
*Eubacterium sp* prevents obesity by BAT activation. **(A)** Regulatory effects of *Eubacterium*_sp on body weight, **(B)** Typical body photographs of mice in different groups. **(C–F)** weights of subcutaneous (Sub), perirenal (PE), and epididymal fat (EP) deposits, and BAT. **(G)** H&E staining images of BAT, **(H)** proteins’ expression associated with BAT, **(I)** proteins’ expression associated with scWAT, **(J,K)** oral glucose tolerance test.and area under the curve (AUC) of oral glucose tolerance test, **(L–O)** Serum TC, TG, LDL-C, HDL-C concentration. **(P–R)** Effects of *Eubacterium sp* on the liver in FHD-fed mice, **(S)** H&E staining images of perirenal fat, **(T)** Daily food consumption in mice.

*Eubacterium* sp., a Gram-positive bacterium of the genus *Eubacterium*, is commonly found in mammalian oral cavities and intestinal tracts. The genus serves as a key taxon in the composition of human intestinal microbiota (Mukherjee et al., 2020). HFD-fed mice were used as study subjects to investigate the role of *Eubacterium* sp. in obesity. Notably, *Eubacterium* sp. significantly reduced body weight gain (Figures 8A, B; Table 4) and fat mass (Figures 8C-F). Histological examination also showed that brown adipose tissue (BAT) from *Eubacterium* sp.-treated mice possessed markedly smaller lipid droplets compared to the Mod group (Figure 8G). The treated group exhibited significant upregulation of UCP1 and OXPHOS proteins in BAT and scWAT, consistent with the overall findings (Figures 8H,I). Additionally, *Eubacterium* sp. supplementation improved glucose homeostasis and blood lipid levels (Figures 8J–O), attenuated hepatic steatosis (Figures 8P–R), and lowered perirenal adipocytes (Figure 8S). These metabolic benefits were independent of food intake (Figure 8T), strongly suggesting that *Eubacterium* sp. reduces adiposity and prevents obesity by activating BAT rather than modulating appetite.

**TABLE 4:** The effect of *Eubacterium sp* on body weight in HFD-fed mice (Mean ± SD).

Groups	Initial weight(g)	End weight (g)	Weight gain (g)	Weight gain rate
Con	20.3 ± 0.2	26.5 ± 1.2	6.2 ± 1.3	30.30%
Mod	20.3 ± 1.1	32.2 ± 0.8	11.9 ± 1.5***	58.84%
Pos	20.4 ± 0.5	27.4 ± 0.9	7.0 ± 1.2***	34.58%
*Eu*-L	20.3 ± 0.5	29.3 ± 1.6	9.0 ± 1.7**	44.38%
*Eu*-M	20.5 ± 0.4	28.3 ± 1.3	7.8 ± 1.5***	38.32%
*Eu*-H	20.5 ± 0.4	26.9 ± 0.9	6.4 ± 1.2***	31.09%

***p < 0.001; **p < 0.01; *p < 0.05; *n* = 6

### 3.9 Margaroleic acid was enriched in serum metabolites

**FIGURE 9:**
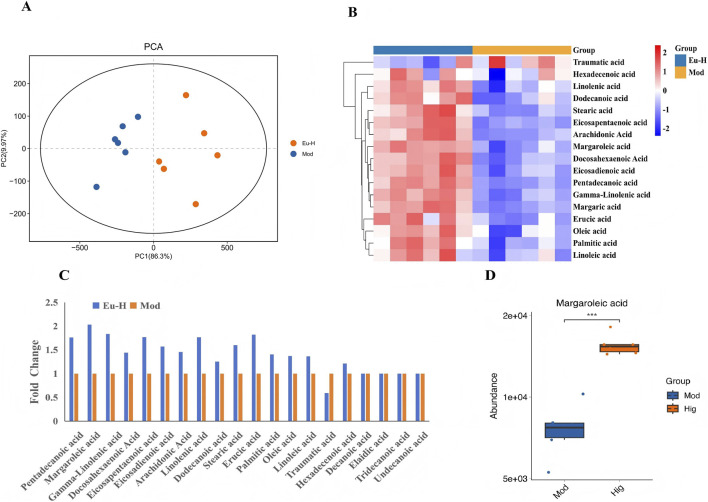
*Eubacterium* sp. administration enriche Margaroleic acid in serum metabolites. **(A,B)** *Eubacterium* sp. treatment alters serum LCFAs in HFD-fed mice. **(C,D)** *Eubacterium* sp. increases Margaroleic acid levels.

Probiotics improve host metabolism via live bacteria and their metabolites (e.g., fatty acids). Our study shows that *Eubacterium* sp. activates BAT and induces WAT browning to prevent obesity, suggesting that its gut metabolites may be a key mechanism in regulating energy metabolism. Given that BAT thermogenesis primarily utilizes long-chain fatty acids (LCFAs) as substrates (Xiang et al., 2018). Consequently, investigating whether *Eubacterium* sp. alters serum levels of long-chain fatty acids is of significant importance. Accordingly, serum samples were subjected to targeted metabolomics to characterize LCFA profiles. The results showed that oral gavage of *Eubacterium* sp. significantly altered the composition of LCFAs (Figures 9A,B). To determine the changes in specific LCFAs after treatment with *Eubacterium* sp., we conducted a detailed analysis of the LCFA levels in mouse serum, comparing those treated with *Eubacterium* sp. to those not treated. Interestingly, the Eu-H group revealed an exceptionally high concentration of margaroleic acid, reaching 2.03 times that observed in the absence of *Eubacterium* sp. (Figures 9C,D). This finding implies that the margaroleic acid produced by *Eubacterium* sp. may play a potential role in activating BAT.

### 3.10 Discussion

Obesity constitutes a pervasive metabolic disorder that actively undermines global health through its associated comorbidities (Kanem et al., 2023). In light of the side effects and high costs linked to conventional pharmacological agents, there is growing interest in exploring natural plant-based alternatives for the management of metabolic diseases (Tak and Lee, 2021). Lotus leaf, widely utilized in traditional medicine as a functional food ingredient (Gao et al., 2025; Guo et al., 2016), has demonstrated various biological activities against obesity and related disorders (Zhang et al., 2023; He et al., 2022; Li et al., 2025). However, the precise molecular mechanisms underlying these effects remain incompletely elucidat.

In this study, dietary supplementation with lotus leaf extract (LLE) significantly attenuated high-fat diet (HFD)-induced body weight gain and reduced fat accumulation in subcutaneous, perirenal, and epididymal adipose tissues. LLE also improved serum lipid profiles—reducing TG, TC, and LDL-C while normalizing HDL-C—and enhanced glucose tolerance during OGTT. Given the central role of insulin resistance in metabolic disturbances, these improvements highlight the potential of LLE in systemic metabolic regulation.

Obesity results from a chronic imbalance wherein energy intake exceeds energy expenditure (Levin et al., 2025). Thus, strategies that enhance energy expenditure represent a promising therapeutic approach. Brown adipose tissue (BAT) is a metabolically active organ that dissipates energy via thermogenesis, and the browning of white adipose tissue (WAT) further contributes to lipid oxidation and energy consumption (Liu et al., 2022). Both BAT activation and WAT browning have been recognized as key mechanisms in improving energy balance and metabolic health (Li et al., 2019; Li et al., 2021; Du et al., 2022; Singh et al., 2021). Our findings reveal that LLE promotes thermogenesis by upregulating UCP1 and OXPHOS proteins (ATP5A1, SDHB, UQCRC2, NDUFB8) in BAT and WAT, confirming enhanced thermogenic capacity at the molecular level.

The anti-obesity effects of LLE were dose-dependent, with higher doses leading to more pronounced reductions in body weight, fat mass, and serum lipids. Correspondingly, UCP1 expression increased progressively with LLE dosage, further supporting the role of BAT activation and WAT browning in its mechanism.

Recent studies have highlighted interactions between BAT activity and gut microbiota (Cani and Hui, 2024; Li et al., 2019). Modulation of gut microbiota has been shown to enhance BAT function and ameliorate obesity in multiple mouse models (Xu et al., 2020; Zhu et al., 2024; Quan et al., 2020; Zeng et al., 2022; Li et al., 2023). In line with this, LLE induced gut microbiota remodeling, notably enriching *Eubacterium* sp. across taxonomic levels. Subsequent intervention with *Eubacterium* sp. reproduced dose-dependent anti-obesity effects similar to LLE, suggesting that this bacterium may mediate part of LLE’s metabolic benefits.

Long-chain fatty acids (LCFAs) contribute to the regulation of energy metabolism by acting as ligands of peroxisome proliferator-activated receptors (PPARs) (Nakamura et al., 2014) and, when produced in brown adipocytes via lipolysis, can activate UCP1 uncoupling in thermogenic fat (Takato et al., 2017). Notably, LCFAs are indispensable for UCP1-mediated uncoupling (Fedorenko et al., 2012) and mediate the primary heat-producing function of BAT (Xiang et al., 2018). Recent evidence further indicates that LCFAs serve as permanently attached substrates of UCP1, facilitating H+ translocation (Fedorenko et al., 2012; Bertholet and Kirichok, 2017; Piel et al., 2021), while short-chain fatty acids lack this co-transport capability.

Probiotics improve host metabolism through both live-microbe-dependent mechanisms and microbe-derived metabolites such as fatty acids. In this context, we hypothesized that LCFAs may mediate BAT activation. Supporting this, treatment with *Eubacterium* sp. significantly elevated serum levels of margaroleic acid, an LCFA. To further investigate the role of margaroleic acid in BAT activation, we plan to systematically validate its effects in future studies: first by treating primary brown adipocytes with various LCFAs *in vitro* and measuring oxygen consumption, followed by *in vivo* administration via oral gavage to assess its regulatory role in BAT function.

We acknowledge certain limitations, including the modest sample size in microbiota analysis and the absence of histopathological evaluation. These aspects warrant further validation in larger cohorts and more comprehensive toxicological studies.

In conclusion, this study demonstrates that LLE alleviates HFD-induced obesity through multi-faceted mechanisms: enhancing BAT thermogenesis, promoting WAT browning, and modulating gut microbiota toward a beneficial composition, potentially mediated by *Eubacterium* sp. and its associated LCFA metabolites. These findings support the potential of lotus leaf as a natural intervention for obesity and provide a mechanistic basis for its further development.

## 4 Conclusion

In conclusion, we integrated data on the gut microbiome, serum metabolome, BAT and WAT. Experimental data indicated that LLE can effectively prevent obesity via gut microbiota-mediated increase in BAT activity and WAT browning. Treatment with *Eubacterium* sp. increased both the population of LCFA-producing bacteria and the production of LCFAs, thereby promoting BAT activation. The present study unraveled the biological mechanisms underlying the protective effect of LLE on obesity, reinforcing its therapeutic potential as a prebiotic in preventing obesity and its metabolic complications.

## Data Availability

The original contributions presented in the study are publicly available. This data can be found at the NCBI repository, accession PRJNA1429535; available at http://www.ncbi.nlm.nih.gov/bioproject/1429535.
