# Supplementary material for: Eubacterium sp. mediates the anti-obesity effect of lotus leaf extract via brown adipose tissue activation and white fat browning
Source: Front Pharmacol. 2026 Mar 9;17:1727610. doi: 10.3389/fphar.2026.1727610 (PMC13006669; doi:10.3389/fphar.2026.1727610)
Supplement: Supplementary file 1 [file Table1.docx]

Table Supplement 1. Qualitative results of the UHPLC-Q-TOF-MS/MS analysis of LLE

| Nub | Rt （min） | Formula | Molecular weight（*m*/*z*） | Fragment ion（*m*/*z*） | Deviation（ppm） | Chemical name |
| --- | --- | --- | --- | --- | --- | --- |
| 1 | 1.252 | C_6_H_8_O_7_ | 191.0198 [M-H] ^-^ | 111.0087 | -6.1 | Citric acid |
| 2 | 1.437 | C_7_H_12_O_6_ | 191.0198 [M-H] ^-^ | 100.9556, 101.2482, 111.0084 | -6.16 | Quinic acid |
| 3 | 5.68 | C_30_H_26_O_12_ | 579.1498 [M+H] ^+^ | 127.0389, 139.0386, 287.0546,409.0896 | -0.17 | Procyanidin B2 |
| 4 | 5.967 | C_30_H_26_O_12_ | 579.1491[M+H] ^+^ | 409.0898, 287.0536, 163.0392, 139.0385,127.0389 | 1.04 | Procyanidin B1 |
| 5 | 6.622 | C_15_H_14_O_6_ | 289.0726 [M-H] ^-^ | 109.0286, 123.0445, 137.0238, 159.0432, 203.0712 | -6.72 | Catechin* |
| 6 | 6.826 | C_15_H_14_O_6_ | 291.0865 [M+H] ^+^ | 123.9437,139.0394, 147.0446 | -0.64 | Epicatechin* |
| 7 | 7.245 | C_21_H_23_O_11_ | 451.2196 [M-H] - | 289.0652 | 1.04 | Catechin-5-glucoside |
| 8 | 9.255 | C_17_H_19_NO_3_ | 286.144 [M+H] ^+^ | 175.0747, 178.0842, 269.1173 | -0.81 | Coclaurine |
| 9 | 9.592 | C_18_H_21_NO_3_ | 300.1594 [M+H] ^+^ | 175.0747, 209.0949, 237.0901, 269.1173 | 0.07 | *N*-methylisococlaurine |
| 10 | 11.073 | C_20_H_25_NO_3_ | 328.1546 [M+H] ^+^ | 297.1304 | -0.81 | *O*-methylarmepavine |
| 11 | 12.077 | C_26_H_28_O_16_ | 595.133 [M-H] ^-^ | 151.004, 178.9982, 271.0267, 300.0287 | -6.12 | Quercetin-3-*O*-xylosyl-glucoside |
| 12 | 12.314 | C_26_H_28_O_12_ | 597.1447 [M+H] ^+^ | 303.0502, 465.1028 | 0.52 | Quercetin-3-*O*-arabinose -galactoside |
| 13 | 12.487 | C_19_H_23_NO_3_ | 314.1749 [M+H] ^+^ | 189.0907, 206.1156, 283.1324 | 0.54 | Armepavine* |
| 14 | 12.891 | C_18_H_21_NO_3_ | 300.1594 [M+H] ^+^ | 107.0494, 145.0642, 189.0908, 252.1143, 268.1086, 283.1323 | 0.07 | *N*-norarmepavine |
| 15 | 14.052 | C_21_H_18_O_13_ | 479.0823 [M+H] ^+^ | 303.0499 | -0.59 | Quercetin-3-*O*-glucuronide* |
| 16 | 14.097 | C_21_H_20_O_12_ | 463.091 [M-H] ^-^ | 300.0291 | -6.03 | Quercetin-3-*O*-galactoside |
| 17 | 14.125 | C_21_H_20_O_12_ | 465.1033 [M+H] ^+^ | 229.046, 303.05 | -1.18 | Isoquercitrin* |
| 18 | 14.787 | C_20_H_18_O_11_ | 433.0796 [M-H] ^-^ | 255.0271, 271.0282, 300.0286 | -4.53 | Avicularin |
| 19 | 15.248 | C_18_H_19_NO_2_ | 282.149 [M+H] ^+^ | 208.0877, 236.083, 251.1064 | -0.51 | *O*-nornuciferine* |
| 20 | 15.864 | C_26_H_32_O_11_ | 519.1876 [M-H] ^-^ | 136.0153, 151.0391, 357.1344 | -0.8 | Pinoresinol-4-*O*-glucoside |
| 21 | 16.275 | C_21_H_18_O_12_ | 463.0882 [M+H] ^+^ | 287.0546 | -2.38 | Kaempferol-3-*O*-glucuronide |
| 22 | 16.325 | C_21_H_20_O_11_ | 449.1079 [M+H] ^+^ | 287.0551 | -0.14 | Kaempferol-3-*O*-glucoside* |
| 23 | 16.387 | C_21_H_20_O_11_ | 449.1078 [M+H] ^+^ | 287.0542, 153.0165 | 0.08 | Cynaroside |
| 24 | 16.942 | C_22_H_20_O_13_ | 491.0855 [M-H] ^-^ | 151.0136,300.0328(100), 315.0528 | -4.85 | Isorhamnetin-7-*O*-glucuronic acid |
| 25 | 17.689 | C_22_H_22_O_11_ | 463.1232 [M+H] ^+^ | 258.0533, 286.0477, 301.0702 | 0.62 | Diosmetin-7-*O-β-D*-glucoside |
| 26 | 20.77 | C_18_H_19_NO_2_ | 282.149 [M+H] ^+^ | 207.0797, 235.0789, 265.1223 | -0.51 | *N*-nornuciferine* |
| 27 | 21.174 | C_19_H_21_NO_2_ | 296.1644 [M+H] ^+^ | 234.1036, 250.0988, 265.1223 | 0.36 | Nuciferine* |
| 28 | 21.521 | C_15_H_10_O_7_ | 301.0369 [M-H] ^-^ | 121.0306, 151.0053, 178.9977, 273.0363 | -5.04 | Quercetin* |
| 29 | 21.992 | C_22_H_20_O_13_ | 491.0855 [M-H] ^-^ | 315.0527 | -4.85 | Isorhamnetin 3-*O*-glucuronide |
| 30 | 22.043 | C_22_H_22_O_12_ | 477.106 [M-H] ^-^ | 299.0213, 314.0441 | -4.5 | Isorhamnetin 3*-O*-glucoside |
| 31 | 22.588 | C_19_H_21_NO_3_ | 312.1596 [M+H] ^+^ | 225.0821, 251.1064, | -0.58 | Pronuciferine |
| 32 | 23.322 | C_15_H_20_O_8_ | 327.2201 [M-H] ^-^ | 121.0689, 165.1287 | -7.32 | Androsin |
| 33 | 23.8 | C_9_H_8_O_4_ | 181.1223 [M+H] ^+^ | 107.085, 117.0764, 135.1157, 145.0946, 163.1074 | 0.03 | Caffeic acid* |
| 34 | 24.373 | C_21_H_20_O_12_ | 465.1176 [M+H] ^+^ | 129.0514, 153.1263, 273.2525 | -0.23 | Myricitrin |
| 35 | 24.625 | C_18_H_26_O_3_ | 291.1953 [M+H] ^+^ | 119.0835, 147.0769, 273.1138 | 0.59 | Oxabolone |
| 36 | 24.961 | C_16_H_18_O_6_ | 307.1901 [M+H] ^+^ | 289.1811, 235.0863 | 0.93 | Cimifugin* |
| 37 | 25.214 | C_16_H_19_NO_3_ | 274.2744 [M+H] ^+^ | 228.234, 256.2629 | -1.26 | 8-*O*-demethylmaritidine |
| 38 | 25.231 | C_19_H_17_NO_3_ | 308.1282 [M+H] ^+^ | 191.0858, 219.0785, 249.0907 | -0.26 | *N*-acetylanonaine |
| 39 | 25.887 | C_16_H_18_O_9_ | 353.2679 [M-H] ^-^ | 135.117, 173.1317, 179.1518, 191.1756 | 2.09 | Cryptochlorogenic acid |
| 40 | 26.359 | C_18_H_28_O_2_ | 277.2165 [M+H] ^+^ | 133.1017, 151.4529, 235.1787 | -1.06 | Bolandiol |
| 41 | 26.712 | C_15_H_16_O_6_ | 293.2105 [M+H] ^+^ | 119.0847, 147.1161 | 2.13 | Cnidimol E |
| 42 | 27.773 | C_18_H_16_O_3_ | 279.1594 [M+H] ^+^ | 238.086, 263.1239 | 0.22 | Magnaldehyde *B* |
| 43 | 28.594 | C_15_H_10_O_6_ | 287.2198 [M+H] ^+^ | 153.1211 | - | Kaempferol* |

Note: The compounds marked with * are the identified standard compounds.
