# Supplementary figures and images for: Eubacterium sp. mediates the anti-obesity effect of lotus leaf extract via brown adipose tissue activation and white fat browning
Source: Front Pharmacol. 2026 Mar 9;17:1727610. doi: 10.3389/fphar.2026.1727610 (PMC13006669; doi:10.3389/fphar.2026.1727610)

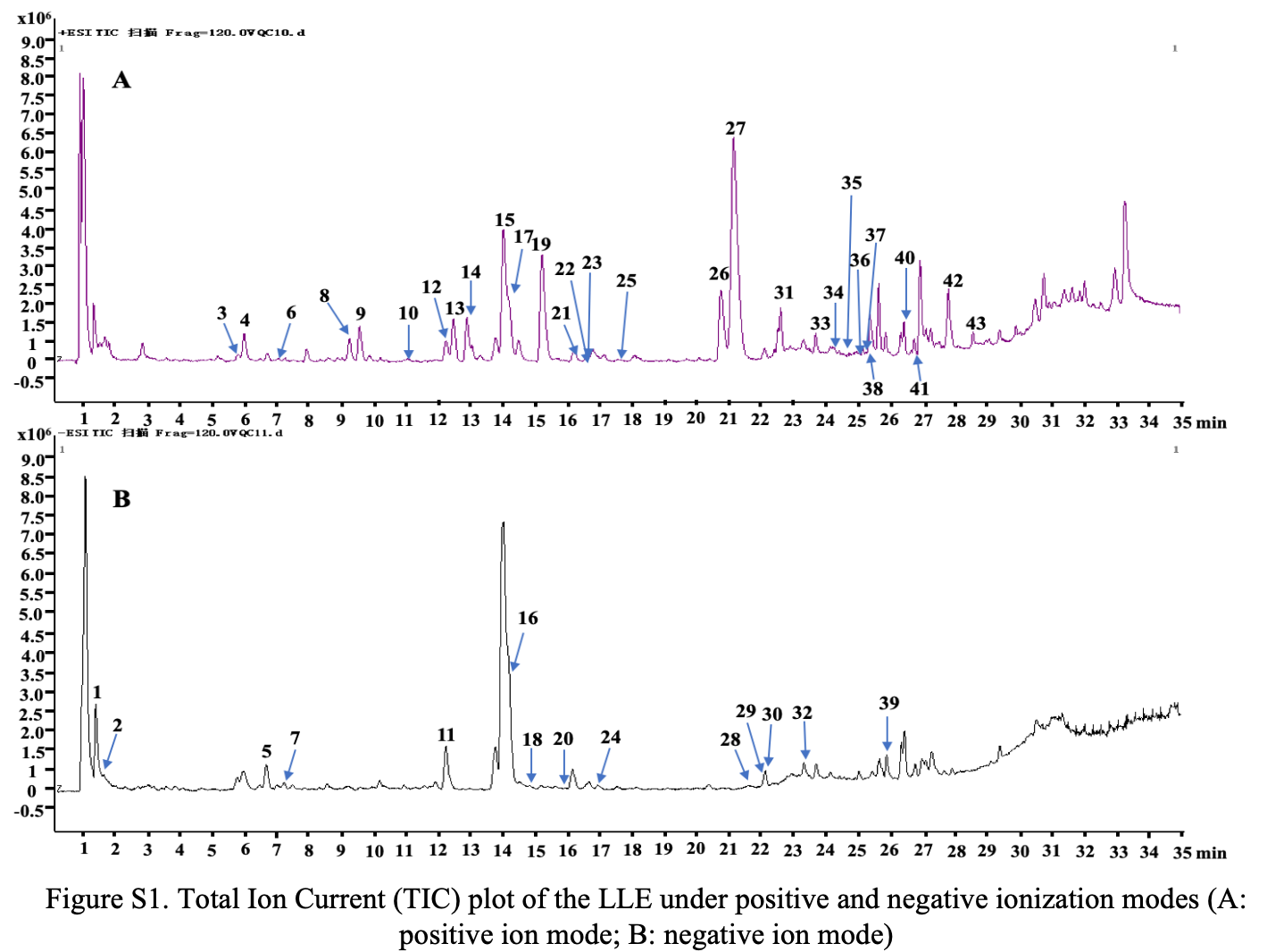

Supplement: Supplementary file 3 [file Image1.png]
